# Supplementary material for: Inheritances, social classes, and wealth distribution
Source: PLoS One. 2021 Oct 27;16(10):e0259002. doi: 10.1371/journal.pone.0259002 (PMC8550378; doi:10.1371/journal.pone.0259002)
Supplement: Attachment — Submitted filename: Response to Reviewers.docx [file pone.0259002.s001.docx]

RESPONSE TO REVIEWERS

*Reviewer #1 comments to the author*

*The paper "Inheritances, social class, and wealth distribution" investigates a model of intergenerational inheritance. The paper is sound and the results are very interesting, in particular, because of the recent discussions on universal income, which certainly would affect wealth distributions.*

*Despite the complexity of the problem, the authors followed the physics tradition of simplifying the system to one subject to only a few forces. Thus, population growth and wealth growth have been ignored in the model, for the sake of simplicity. The authors also considered that, on average, the ratio of male/female is one, and only same-gender*

*marry is allowed. It would be interesting to see how the same gender marries and differences in the ratio of male/female could affect the intergenerational distribution of wealth.*

*Despite the simplicity of the model, the authors conclude that even in a society with uniform wealth distribution would become unequal after few generations if the number of children and marriage among people from different classes is less likely than marriage between couples from the same class.*

*I enjoyed a lot reading this paper and the paper has the merit to be published in PLOS ONE. My only suggestion to the authors is to include a paragraph on the discussion to highlight the limitations of the model and how it could be improved by including population and wealth growth.*

As suggested by the referee, we have rewritten the conclusion. After summarizing the power-law behavior obtained for the wealth distribution of a society with multiple classes, we wrote a completely new paragraph to highlight the limitations of our model:

*"The exponents reported here differ from the ones observed empirically, that typically range from 1.5 and 3 [2, 3]. Also, note that, in our model, the power-law behavior is observed for intermediate values of wealth and not in the tail for the wealthier. The goal here was to focus on the effect of inheritances and social classes on the wealth distribution. Obviously other factors are also at play and these should be considered in future studies for a more detailed model."*

We also added additional paragraphs to discuss three possible follow up studies for more realistic descriptions. The 1st one considers the inclusion of an underlying social network, a hypothesis that was already discussed in our 1st submission. The 2nd one considers the inclusion of a lifecycle model, in the lines of the proportional random growth models, and allowing for wealth grow:

*"We also assumed that the sum of the wealth of all individuals remain constant, and that each married couple transmits to its offspring exactly its entire received inheritance. Our model is purely intergenerational. Any wealth evolution during lifecycles is neglected. It would then be interesting to couple our model to lifecycle models, like for example the ones in which the evolution of the wealth of individuals are proportional to their initial wealth, but with a random proportionality constant (the proportional random growth models mentioned in the introduction [8–13]). The global wealth evolution depends in this case on the probability distribution of this proportionality constant. If some small wealth dissipation is included, these models also predict power-law behaviors, with α > 1 [7]."*

In the last paragraph, we discuss the possibility of having population growth:

*"In our analysis, we used fixed offspring distributions, that did not change at each new generation. From an historical perspective, we know this is certainly not the case for any society in our world. Moreover, we mostly considered offspring distributions that preserved, at least approximately, the total population of the society. These offspring distributions led to the stationary wealth distributions we have described in our article. An investigation with variable offspring distributions, allowing the evolution of the population, is straightforward within our model and may also be envisaged in the future."*

We thank Reviewer #1 for all comments and suggestions.

*Reviewer #2 comments to the author*

*The paper proposes a model to investigate the impact of inheritance on wealth distribution, considering that wealth is a finite and constant resource equally divided among the offspring over the generations.*

*The results of the model are interesting and non-trivial.*

*However, keeping the wealth constant seems to be out of the real situation (new wealth are constantly created), which compromises any possibility of comparing the model's*

*results with the empirical values.*

*Too bad because the model could achieve something closer to the real world with one or two more considerations.*

*For example, introducing the fact that an individual born poor can become rich (with a given probability - power law?), or the opposite, and following including new wealth to the system.*

We thank the referee for the comments. In this article, and as clearly stated now in the conclusion, we wanted *"to focus on the effect of inheritances and social classes on the wealth distribution"*. We agree that this is a simple model that neglects important aspects of the wealth evolution. In particular, and as pointed out by the referee, wealth may be created (or destroyed), and each individual is capable of investing their capital during their lifetime, increasing (or reducing) the wealth they received from inheritance. Nevertheless, it is important to isolate the contribution of each of these aspects to the wealth distribution. We are convinced that the introduction of these aspects in our model would improve it considerably, and we discuss now how this could be done in a follow up study. As mentioned before in the answer of reviewer 1, we included the following paragraph in the conclusion:

*"We also assumed that the sum of the wealth of all individuals remain constant, and that each married couple transmits to its offspring exactly its entire received in- heritance. Our model is purely intergenerational. Any wealth evolution during lifecycles is neglected. It would then be interesting to couple our model to lifecycle models, like for example the ones in which the evolution of the wealth of individuals are proportional to their initial wealth, but with a random proportionality constant (the proportional random growth models mentioned in the introduction [8–13]). The global wealth evolution depends in this case on the probability distribution of this proportionality constant. If some small wealth dissipation is included, these models also predict power-law behaviors, with α > 1 [7]."*

*It is written briefly in the introduction and conclusion sections that the empirical evidence suggests the exponent alpha varies between 1.5 and 3. However, details are not discussed (for instance, why these values? What is the reason for such dispersion in the values, etc.).*

*I suggest creating a new section (e.g. discussion section) exploring empirical evidence and how your model could be compared to them?*

*That is, in what way to consider constant wealth compromise the quantitative outcome if compared to the empirical evidence?*

*For instance, the model predicts an exponent ~ 2/3 that is much smaller than the*

*inferior limit of the empirical result.*

In what regards recent empirical evidence, reference [3] is particularly comprehensive, and shows (corroborating the results from the data already collected by Pareto in [1]) that this exponent exists approximatively in real societies and varies between 1.5 and 3. These exponents translate the societies' wealth inequality. As explained in our introduction, *"many models have been proposed to explain the tail distribution of wealth [8–11] (or, more recently [12, 13], in the context of physics), mainly along the lines of proportional random growth"*. In the conclusion, the comparison between our results and the empirical ones is now highlighted in a paragraph. The following paragraphs discuss how the introduction of new aspects of the model may influence the value of the power-law exponent alpha. First, by having a social network with a different underlying topology. Next, by linking our intergenerational model to the proportional random growth models already mentioned in the introduction. These are mainly phenomenological. The predicted power-law exponent alpha depends on the particular distribution of the proportionality constant and on the details of the wealth dissipation mechanism each model presents, but it is always larger than 1. The interested reader is referred to reference [7], which makes a very interesting review of several different models of this type.

*Other suggestions:*

*Fig. 7 : I suggest including the value of the power-law exponent in the caption and stress in this caption the robustness of this value. That is an important finding, and it must be clear to the reader.*

We followed the suggestion made by the referee.

*- I became curious to see two more graphs about the Gini coefficient that could give more insights to the interpretation of the results: i) G as a function of beta, and ii) G as a function of Nc. You cited these things in the paper, but I think it is more appropriated to present them in a graph/figure.*

We thank the referee for the suggestion. The two graphs follow below:

The tables containing the data for these plots are here

| Left figure | Nc=3 | Nc=10 |
| --- | --- | --- |
| Beta | Gini | Gini |
| 0 | 0,35 | 0,35 |
| 1 | 0,46 | 0,72 |
| 2 | 0,56 | 0,82 |
| 5 | 0,66 | 0,85 |
| 10 | 0,68 | 0,87 |

| Right figure | Beta=1 | Beta=10 |
| --- | --- | --- |
| Nc | Gini | Gini |
| 1 | 0,35 | 0,35 |
| 3 | 0,46 | 0,68 |
| 10 | 0,72 | 0,87 |
| 20 | 0,82 | 0,92 |
| 50 | 0,89 | 0,95 |
| 100 | 0,93 | 0,95 |

These are the Gini's coefficients associated with the limiting stationary wealth distribution. Since the evolution of the wealth distribution is a random process, the final limiting Gini's coefficient oscillates around its average value. Thus, there is an associated error that we tried to minimize, at least below 0,01.

So, for the simpler case in which Nc=1, Gini's coefficient evolution is

Determining the average and mean standard deviation from the ensemble of values from generation 10 to 30 gives: Gini=0,354+-0,001.

Part of this figure was presented in the article (from generation 0 to 10), in the inset of figure 2.

Other cases may have stronger oscillations, as for instance the case Beta=5, Nc=10:

In this case, determining the average and mean standard deviation from the ensemble of values from generation 50 to 100 gives: Gini=0,852+-0,008.

Although all this information is interesting, we think the two graphs of Gini vs Beta and Gini vs Nc do not present a major achievement: they show a tendency that is already described in the text. So, for the sake of clarity and objectivity, we prefer not to include them in our article, to focus on the general features of the limiting wealth distribution, as reported in our conclusion.
